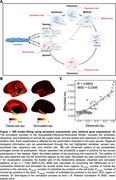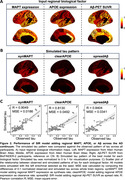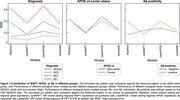# Impact of regional MAPT, APOE, and Aβ on tau propagation in Alzheimer’s disease: Insights from a connectome‐based simulation model

**DOI:** 10.1002/alz.094588

**Published:** 2025-01-09

**Authors:** Yu Xiao, Olof Strandberg, Vincent Bazinet, Golia Shafiei, Hamid Behjat, Nicola Spotorno, Ruben Smith, Danielle van Westen, Sebastian Palmqvist, Niklas Mattsson‐Carlgren, Erik Stomrud, Bratislav Misic, Alain Dagher, Oskar Hansson, Jacob W. Vogel

**Affiliations:** ^1^ Department of Clinical Sciences Malmö, SciLifeLab, Lund University, Lund Sweden; ^2^ Clinical Memory Research Unit, Department of Clinical Sciences Malmö, Faculty of Medicine, Lund University, Lund Sweden; ^3^ McConnell Brain Imaging Centre, Montréal Neurological Institute, McGill University, Montréal, QC Canada; ^4^ Penn Lifespan Informatics and Neuroimaging Center, University of Pennsylvania, Philadelphia, PA USA; ^5^ Department of Psychiatry, Perelman School of Medicine, University of Pennsylvania, Philadelphia, PA USA; ^6^ Department of Diagnostic Radiology, Clinical Sciences, Lund University, Lund Sweden; ^7^ Memory Clinic, Skåne University Hospital, Malmö Sweden; ^8^ Department of Neurology and Neurosurgery, McGill University, Montréal, QC Canada

## Abstract

**Background:**

Tau pathology, a hallmark of Alzheimer’s disease (AD), is thought to spread cell‐to‐cell via axonal connections, beginning focally before expanding throughout the brain. This study uses computational models to investigate the interplay between network spread and regional vulnerability in influencing tau spread, focusing specifically on MAPT and APOE genes, and Aβ plaques.

**Method:**

66 regional (Desikan‐Killiany atlas) tau‐PET standardized uptake value ratio (SUVR) values were extracted from participants in the Swedish BioFINDER‐2 study: 429 cognitively normal (CN), 91 subjective cognitive decline (SCD), 168 mild cognitive impairment (MCI), and 182 AD. Values were adjusted for mean choroid plexus signal and converted to tau‐positive probabilities using two‐component Gaussian mixture models. The Susceptible‐Infectious‐Recovered (SIR) model (Fig. 1A) was employed to simulate tau spread through brain networks measured using structural connectivity from young individuals. We examined the roles of MAPT, APOE, and Aβ in tau propagation by parameterizing them regionally to influence tau synthesis, clearance, spreading, or misfolding. Regional MAPT and APOE were extracted from Allen Brain Atlas, and Aβ from Aβ‐PET. Performance of both baseline models (connectivity‐only) and models incorporating regional biological information were evaluated based on their ability to reconstruct observed regional tau levels. Performance was evaluated across the whole sample and groups based on diagnosis, APOE e4 carriage and Aβ positivity.

**Result:**

The SIR model recapitulates observed tau patterns, suggesting connectivity‐based propagation in early‐stage regions (Fig. 1B, C). Allowing regional MAPT to moderate normal tau synthesis improved the model fit overall, and for all groups except CN or Aβ‐ participants (Fig. 2,3). Regional APOE or Aβ information did not enhance the model performance overall (Fig. 2B, C). However, allowing regional APOE to moderate tau clearance showed better performance in APOE e4 carriers vs. non‐carriers, and allowing regional Aβ to moderate tau spread improved performance compared to baseline model among Aβ+ individuals (Fig. 3).

**Conclusion:**

Our results suggest that brain connectivity explains early temporal lobe tau spread patterns, while regional intrinsic (MAPT) and disease‐related (Aβ) susceptibility may influence spread of tau into other regions at later stages. Future work will test other hypotheses of tau spread by refining this model with additional biological information.